# Influence of Exercise Mode on Post-exercise Arterial Stiffness and Pressure Wave Measures in Healthy Adult Males

**DOI:** 10.3389/fphys.2018.01468

**Published:** 2018-10-17

**Authors:** Doris R. Pierce, Kenji Doma, Hayleigh Raiff, Jonathan Golledge, Anthony S. Leicht

**Affiliations:** ^1^Sport & Exercise Science, James Cook University, Cairns, QLD, Australia; ^2^Sport & Exercise Science, James Cook University, Townsville, QLD, Australia; ^3^Department of Health and Sport Science, University of Dayton, Dayton, OH, United States; ^4^Queensland Research Centre for Peripheral Vascular Disease, James Cook University, Townsville, QLD, Australia; ^5^Department of Vascular and Endovascular Surgery, The Townsville Hospital, Townsville, QLD, Australia

**Keywords:** carotid-femoral pulse wave velocity, augmentation index, vascular hemodynamics, aerobic exercise, resistance exercise

## Abstract

**Background:** Exercise mode has been reported to be an important determinant of arterial stiffness and wave reflection changes following a brief bout of exercise with inconsistent results to date. This study examined the impact of exercise mode on arterial stiffness and pressure wave measures following acute aerobic exercise (AER), resistance exercise (RES), and a control (CON) condition with no exercise.

**Methods:** In a randomized, cross-over, repeated measures design, 21 healthy adult males (26.7 ± 7.2 years) undertook three experimental intervention sessions: AER (30-min cycle ergometry at 70–75% maximum heart rate), RES (3 × 10 repetitions of six upper and lower body exercises at 80–90% of 10-repetition maximum) and CON (30-min seated rest). Measures of arterial stiffness and pressure waves, such as carotid-femoral pulse wave velocity (cf-PWV), augmentation index (AIx), AIx corrected for heart rate of 75 (AIx75), and forward wave (Pf), backward wave (Pb) and reflection magnitude, were assessed at Rest and at 10-min intervals for 60 min after the intervention sessions. Comparisons between interventions and over time were assessed via repeated measures ANOVA and *post-hoc* Tukey's tests.

**Results:** No significant differences in cf-PWV were noted between the three interventions at rest or post-intervention. However, RES led to significantly greater post-intervention AIx, AIx75, Pf, and Pb compared to AER and CON with AIx75 also remaining significantly elevated throughout the post-intervention period. In contrast, AER resulted in a brief, significant elevation of AIx75 and no change in cf-PWV, Pf, Pb, and reflection magnitude.

**Conclusions:** Exercise mode, specifically RES and AER, significantly influenced the time course of pressure wave reflection responses following a brief bout of exercise in healthy adult males. Distinct adjustments during exercise including changes in blood pressure and vasomotor tone may be key modulators of post-exercise arterial function. Identification of modal differences may assist in understanding the impact of exercise on cardiovascular function and the mechanisms by which exercise benefits vascular health.

## Introduction

Increased functional, that is endothelium-mediated (Zieman et al., 2005), arterial stiffness measured via carotid-femoral pulse wave velocity (cf-PWV) has been reported to be highly predictive of the future risk of cardiovascular events (Mattace-Raso et al., 2006) with a 1 ms^−1^ increase in cf-PWV associated with a 10% increase in cardiovascular event risk (Vlachopoulos et al., 2010b). The assessment of arterial stiffness via cf-PWV is particularly effective in identifying cardiovascular event risk in older subjects, whereas the assessment of pressure wave reflection and left ventricular afterload via augmentation index (AIx), has been suggested as a diagnostically more useful indicator of future cardiovascular event risk in younger individuals (McEniery et al., 2005). Augmentation index is influenced by both timing and magnitude of the reflected wave with contemporary wave form analysis also allowing the quantification of reflection magnitude and its individual components, forward and backward waves (Westerhof et al., 2006). Several studies have suggested that greater aortic wave reflection, as indicated by elevated AIx/reflection magnitude, adversely affects left ventricular systolic loading and coronary perfusion with their pathological significance demonstrated in cardiovascular (Vlachopoulos et al., 2010b) and several other diseases (London et al., 2001; Chen et al., 2010; Russo et al., 2011). In addition to their ability to predict cardiovascular event risk, these indices have been reported to be predictive of the success of different therapies, such as hypertensive treatments (Mahmud and Feely, 2008). Cardiovascular medications including beta-blockers have been reported to reduce cf-PWV and wave reflection (Mahmud and Feely, 2008), most likely due to reductions in sympathetic activity (Harvey et al., 2017). Additionally, exercise has been shown to reduce both arterial stiffness (i.e., cf-PWV; Kingwell et al., 1997; Heffernan et al., 2007a) and wave reflection (i.e., AIx and reflection magnitude; Munir et al., 2008; Millen et al., 2016). However, such exercise-induced changes may be influenced by exercise mode with several studies reporting variable results.

Our recent systematic review and meta-analysis of 45 studies (Pierce et al., 2018) suggested that exercise mode was an important determinant of arterial stiffness and wave reflection changes following a brief bout of exercise with distinct responses evident following acute aerobic (AER) and resistance (RES) exercise. However, only two previous studies (Heffernan et al., 2007a; Collier et al., 2010) have directly compared exercise-induced changes in arterial stiffness (i.e., cf-PWV) following different exercise modes with discrete responses following each mode identified. To date, no studies have examined changes in both arterial stiffness and wave reflection indices following a bout of exercise of varying mode including a control protocol of no exercise. Additionally, as post-exercise changes in arterial stiffness and wave reflection can be short-lived (Mutter et al., 2016), infrequent or isolated monitoring undertaken previously (Heffernan et al., 2007a) may have failed to detect changes. Simultaneous assessment of cf-PWV, AIx and wave reflection measures has been recommended to obtain a comprehensive account of exercise-induced changes (Laurent et al., 2006). Consequently, the aim of this study was to examine the influence of an acute bout of AER and RES, as prescribed for health (Garber et al., 2011), on the time-course of post-exercise changes in indices of arterial stiffness and wave reflection. Based upon the results of our meta-analysis (Pierce et al., 2018), we hypothesized that AER would have a minimal (cf-PWV) or reducing (AIx) effect while RES would result in an unfavorable, increasing effect. Identifying the potential beneficial or adverse effects of different exercise modes on arterial stiffness and wave reflection may assist in understanding the impact of exercise on cardiovascular function and health.

## Methods

### Participants

Twenty-one healthy, adult males aged between 18 and 43 years were recruited for this study. Volunteers provided written informed consent in accordance with approval by the James Cook University Human Research Ethics Committee (H5021, H6733). The recruited participants were recreationally active with experience in both aerobic and resistance exercise (~3–4 times/week, >3 months); no trained athletes were included in the cohort. All participants completed a pre-participation medical history questionnaire. Exclusion criteria included a history of cardiovascular disease, more than one cardiovascular disease risk factor (Swain et al., 2014), resting hypertension (systolic blood pressure, BP >140 mmHg, diastolic BP > 90 mmHg), any prescription medication use, and currently smoking. Women were excluded from the present study to minimize factors that influence arterial stiffness responses as differences have been noted between sexes (Doonan et al., 2013), and with different phases of the menstrual cycle (Madhura and Sandhya, 2014).

### Study design

The study was constructed as a randomized, cross-over, repeated-measures intervention. Participants attended a familiarization session followed by three, separate, intervention sessions consisting of one bout of AER, RES or no exercise (CON). There was a minimum of 72 h between sessions (Heffernan et al., 2007a), and each session consisted of initial rest, experimental intervention, and recovery. During the familiarization session, anthropometric data and exercise workloads for each participant were determined.

Intervention sessions consisted of either 30 min seated rest (CON), 30 min of cycling at 70–75% of age-predicted maximum heart rate (HR_max_, AER) or ~30 min of resistance exercise (RES) consisting of 3 sets × 10 repetitions of squat, chest press, leg curl, prone row, shoulder press, and biceps curls. The AER and RES interventions were designed to represent typical sessions in accordance with current guidelines for improvement and maintenance of cardiovascular health (Garber et al., 2011) rather than being matched for intensity, workload, or muscle group exercised, which is near impossible for these modes. All sessions took place in a controlled environment (mean ± SD, temperature 22.7 ± 1.6°C, humidity 64.7 ± 7.2%, barometric pressure 1,016.6 ± 2.4 mbar), and intervention sessions were randomly allocated using an online program (http://www.randomizer.org/). Participants were blinded to the order of the experimental interventions until arrival at the laboratory. All sessions were conducted in the morning with each participant performing sessions at the same time of day to minimize any potential diurnal variation. Participants were instructed not to ingest any food or drink, except water, after midnight before the sessions, and to avoid alcohol, caffeine and exercise for at least 24 h preceding each session (Laurent et al., 2006).

### Familiarization session

Participants reported to the laboratory for their individual familiarization session, where height was recorded via a wall-mounted stadiometer (SECA, Hamburg, Germany), and mass, body fat percentage, and body mass index were assessed using bioelectrical impedance scales (Tanita BC-545N, Tanita Corporation of America, Arlington Heights, IL, USA). Following anthropometric data collection, participants were fitted with a heart rate monitor (RS800, Polar Electro, Kempele, Finland) and lay supine on a cushioned examination table for the consecutive measurement of brachial BP, cf-PWV, and wave reflection measures via pulse wave analysis. Participants then completed a bout (6–9 min) of AER on a cycle ergometer (828E, Monark, Varberg, Sweden) to determine the workload required to achieve their individual target HR (70–75% of age-predicted HR_max_) for the AER intervention. Age-predicted HR_max_ was calculated using the equation of Inbar, which has been recommended as an accurate estimation of HR_max_ (Inbar et al., 1994; Robergs and Landwehr, 2002).

Within 5 min of the bout of AER, participants completed a brief warm-up of standardized dynamic stretches, followed by a 10-repetition maximum (10-RM) protocol for back squat, chest press, leg curl, shoulder press, prone row, and biceps curl. The chest press, shoulder press, and leg curl protocols were conducted using a multi-station training apparatus (Nautilus International, Independence, VA, USA), while back squats were conducted using a Smith machine (MPL 706, Maxim Fitness, Australia); prone row and biceps curls were performed using free weights (i.e., bar and plates). The load for each exercise was determined as previously described (Doma et al., 2015). Briefly, participants completed an initial set of ten repetitions at 50% of their estimated 10-RM. Based upon participants' self-perceived, estimated repetitions-to-failure, weight was progressively increased until the participant could just achieve ten repetitions. To minimize fatigue, the load was adjusted by 5–10% if participants perceived it as excessively heavy or light by the 5th repetition, were unable to complete a set, or did not achieve maximal effort by the tenth repetition. A rest period of 2–3 min was allowed between each set and exercise. All participants achieved their 10-RM within 3–5 attempts.

### Intervention sessions

Participants initially undertook 20-min of supine rest on a cushioned examination table with HR recorded (RS800, Polar Electro, Kempele, Finland) each minute during the final 10 min. Subsequently, brachial BP was recorded via a Connex®ProBP™ 3400 digital BP device (Welch Allyn, Mississauga, Ontario, Canada) followed by assessment of cf-PWV and pulse wave analysis measures using a semi-automated system (Xcel, AtCor Medical Pty Ltd, West Ryde, Australia) following previously described guidelines (Van Bortel et al., 2012).

The AER session consisted of 30 min of cycling on an ergometer (828E, Monark, Varberg, Sweden) at 65–70 revolutions per minute at the pre-determined workload. Rating of perceived exertion (RPE, 6–20 category scale; Borg, 1982) and HR were recorded every minute. For RES, participants performed a warm-up set of six repetitions at 50% of their pre-determined 10-RM workload. Immediately following the warm-up set, participants completed three working sets of 10 repetitions of each exercise (i.e., 90% of 10-RM for squat and prone row, 80% of 10-RM for biceps curl, and 80–90% of 10-RM for chest press, shoulder press and leg curl in this order) with 1 min of rest between each set and each exercise. Participants completed each RES exercise in ~5 min, after which HR and RPE were recorded. All participants completed RES in ~30 min. During CON, the participant sat in a chair for 30 min maintaining good posture and keeping both feet on the floor with HR recorded every minute. Comparable exercise responses for each intervention were determined via analysis of HR and RPE values at 5-min intervals (i.e., Stages 1–6). Upon completion of each intervention, participants immediately returned to the examination table and recovered in the supine position for 60 min. Recordings of brachial BP, cf-PWV and pulse wave analysis measures were obtained at 10-min intervals post-intervention, starting at 10 min.

### Central and peripheral cardiovascular measures

Brachial BP measurements (systolic and diastolic BP, mean arterial pressure) were assessed using the automated digital BP device described above. Assessments of cf-PWV were conducted in accordance with previously described guidelines (Van Bortel et al., 2012) using a semi-automated system (Xcel, AtCor Medical Pty Ltd, West Ryde, Australia). The system uses a partially inflated femoral cuff together with carotid applanation tonometry for a time efficient and convenient assessment (Hwang et al., 2014) with the system (i.e., pressure responses) calibrated in accordance with manufacturer's instructions. Oscillometric pulse wave analysis has been shown to be a valid and reliable estimate of central BP, AIx (absolute and corrected for a HR of 75 bpm, AIx75) responses during resting (Hwang et al., 2014) and post-exercise (Lim et al., 2016) conditions. The participant's right carotid and femoral artery were located and marked for repeated measurements. The thigh cuff was placed 15 cm distally to the location of the femoral artery, and the distance between carotid artery and top edge of the thigh cuff measured in a straight line with a metal measuring tape. Eighty per cent of the carotid-femoral distance (measured distance−15 cm) was used in the calculation of cf-PWV, as previously recommended (Van Bortel et al., 2012). Assessment of cf-PWV included the automatic inflation/deflation of the femoral cuff with 10 s of simultaneous and valid signals from the tonometer at the carotid artery and femoral cuff recorded (i.e., in-built automatic quality control feature). Upon completion of cf-PWV assessment, the module was disconnected from the femoral cuff and reconnected to the cuff placed on the participant's right arm to perform a pulse wave analysis assessment. The following measures were obtained from the pulse wave analysis: HR, pulse pressure, augmentation pressure, AIx, and AIx75. Wave reflection measures were obtained via wave separation analysis using the SphygmoCor CVMS software (AtCor Medical, Sydney, Australia) and consisted of forward (Pb) and backward (Pb) pressure waveforms, and reflection magnitude ([Pb/Pf] × 100) (Westerhof et al., 2006). Assessment of reflection magnitude incorporated a triangular flow estimate to calculate the forward and backward components of the aortic wave (Westerhof et al., 2006) and was based on measured pressure alone with the measurement of flow waves unnecessary. The SphygmoCor Xcel system has been reported to produce valid and highly reliable measurements of cf-PWV, AIx/AIx75 and reflection magnitude in healthy and diseased populations (Hwang et al., 2014; Stoner et al., 2017). In our laboratory, the technical error of measurement for cf-PWV, AIx, AIx75, Pf, Pb, and reflection magnitude during rest in young healthy adults were 0.3 ms^−1^, 4.0, 4.6%, 3.9 mmHg, 2.1 mmHg and 7.9%, respectively.

### Data and statistical analyses

Based upon a previous study reporting a 0.5 ms^−1^ decrease in cf-PWV as statistically significant (medium effect size = 0.58) following a bout of exercise (Heffernan et al., 2007a), an *a priori* power analysis suggested that 16 participants were required to detect significant differences between interventions (power = 80%, *p* < 0.05). All data were collated and/or analyzed using Microsoft Excel (v15.29.1, Microsoft Corporation, Redmond, Washington, United States) and Statistical Package for the Social Sciences version 23 (IBM, Armonk, New York, United States) software with results presented as mean ± SD. Due to technical issues, missing data (~4–5%) were evident during the study and subsequently replaced by averaging values before and after the missing value for the relevant variable prior to analysis.

Comparisons between interventions (CON vs. AER vs. RES) over time (Rest vs. post-intervention time points) for cf-PWV and pulse wave analysis variables were examined via two-way (intervention × time), repeated measures analysis of variance (ANOVA). Specifically, cf-PWV and pulse wave analysis variables were assessed via a 3 × 7 (intervention × time) two-way, repeated measures ANOVA, while exercise HR and RPE for each 5-min stage were analyzed by a 3 × 6 and 2 × 6 (intervention × time) two-way, repeated measures ANOVA, respectively. Session differences for carotid-femoral distance, environmental conditions (temperature, humidity, barometric pressure), average resting HR, and average exercise HR were analyzed using a one-way repeated measures ANOVA. Where necessary, all *post-hoc* comparisons were conducted via Tukey's HSD tests. Statistical significance was set at *p* < 0.05.

## Results

Participant demographic characteristics were as follows (mean ± SD): age 26.7 ± 7.2 years, height 1.77 ± 0.06 m, body mass 82.4 ± 10.3 kg, body fat 17.3 ± 5.9%, resting HR 55.1 ± 0.5 bpm, and HR_max_ 188 ± 5 bpm.

### Responses during intervention

A significant intervention-time interaction was evident for HR with values significantly greater for RES compared to AER at most stages and values significantly greater compared to CON at all stages (Table 1). There were no intervention-time interactions for RPE, but a main effect of mode was evident with greater RPE for RES compared to AER (Table 1).

**Table 1:** Cardiovascular and perceptual responses during aerobic (AER), resistance (RES), and no (CON) exercise interventions (*n* = 21).

		**Stage 1**	**Stage 2**	**Stage 3**	**Stage 4**	**Stage 5**	**Stage 6**	**Main effect of intervention *P* < 0.05**	**Main effect of time *p* < 0.05**
**HR** (bpm)	AER RES CON	129.4 ± 9.2^*^ 156.2 ± 14.6^*^^†^ 63.8 ± 7.3	139.1 ± 9.2^*^ 147.5 ± 19.5^*^ 63.5 ± 9.5	139.0 ± 7.2^*^ 141.1 ± 16.7^*^ 63.4 ± 8.9	138.6 ± 8.2^*^ 148.9 ± 16.9^*^^†^ 62.8 ± 8.0	139.3 ± 8.0^*^ 160.8 ± 11.7^*^^†^ 62.5 ± 8.5	137.7 ± 7.8^*^ 152.5 ± 16.4^*^^†^ 62.5 ± 7.7	RES > AER > CON	Stage 6 > 3
**RPE**	AER RES CON	12.9 ± 2.2 18.0 ± 2.0 n/a	13.6 ± 2.1 17.6 ± 2.3 n/a	13.7 ± 1.9 17.1 ± 2.5 n/a	13.9 ± 1.8 18.6 ± 2.0 n/a	13.8 ± 1.8 18.2 ± 2.4 n/a	13.5 ± 2.0 18.2 ± 2.8 n/a	RES > AER	Stage 4 > 1, 2, 3

*Data are expressed as mean ± SD. HR, heart rate; AER; aerobic exercise; RES, resistance exercise; CON, no exercise; bpm, beats per minute; RPE, rating of perceived exertion; n/a, not assessed*.

* *p < 0.05, significantly different to CON within stage*.

† *p < 0.05, significantly different to AER within stage*.

### Rest-to-post intervention responses

#### Pulse wave velocity

**Figure 1:**
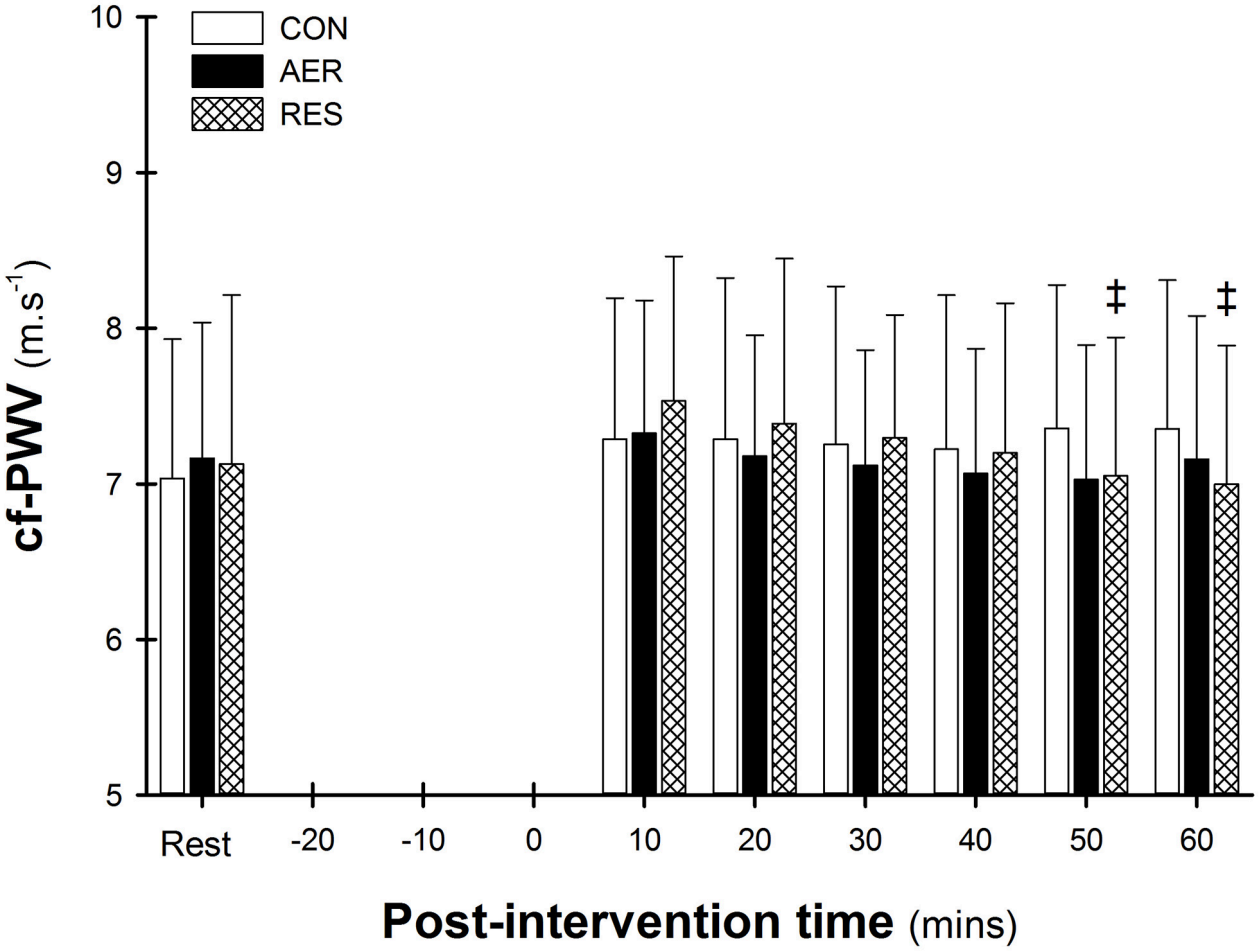
Carotid-to-femoral pulse wave velocity (cf-PWV) at rest and post-intervention. Data are presented as mean ± SD. ^‡^*p* < 0.05 significantly different to 10 min (intervention-time interaction).

A significant intervention-time interaction was identified with significantly lower cf-PWV at 50 and 60 min compared to 10 min post-intervention following RES (Figure 1). There were no significant differences between interventions at any post-intervention time point. A significant main effect of time was evident with the initial (10 min) post-intervention value greater than Rest and 50 min (7.38 ± 0.89 vs. 7.11 ± 0.94 and 7.15 ± 0.89, *p* < 0.05; Figure 1).

#### AP, AIx, and AIx75

**Figure 2:**
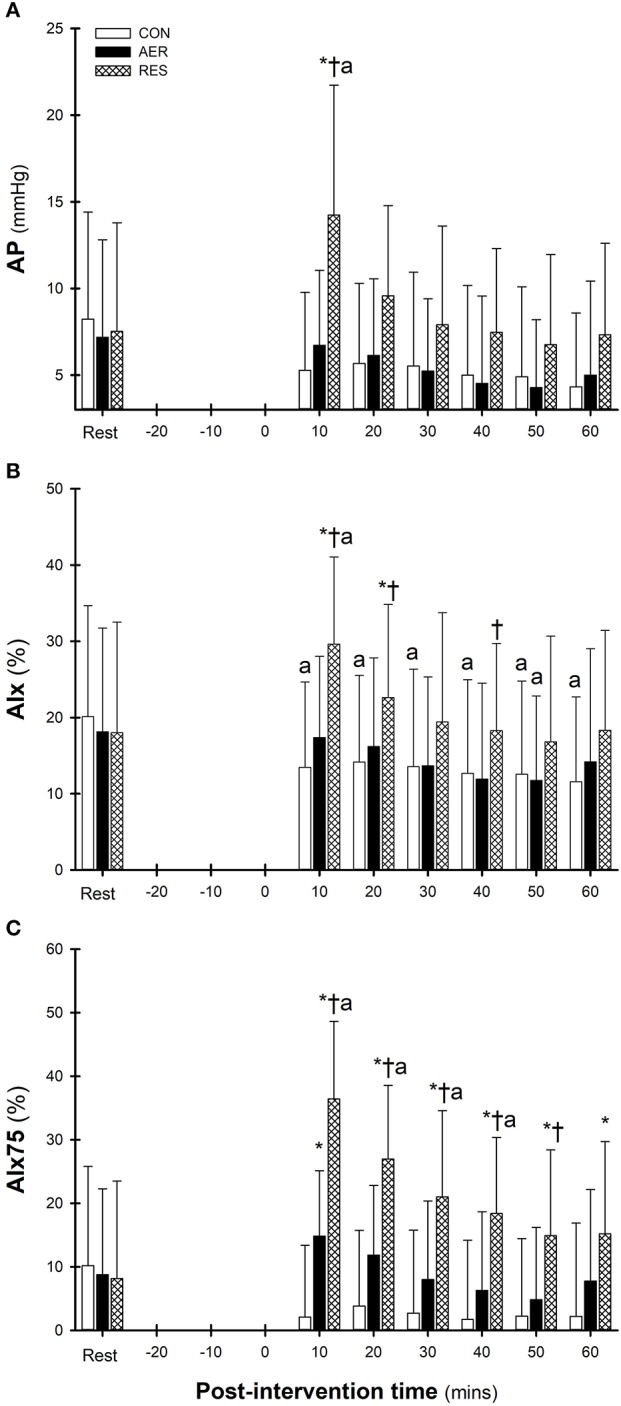
Augmentation pressure (AP, **A**), augmentation index (AIx, **B**), and augmentation index corrected to a heart rate of 75 (AIx75, **C**) at Rest and throughout the post-intervention. Data are presented as mean ± SD. ^a^*p* < 0.05 significantly different to REST within intervention; *p < 0.05 significantly different to CON (intervention-time interaction); *p* < 0.05 significantly different to AER (intervention-time interaction).

Measures for AP, AIx and AIx75 followed similar post-intervention patterns (Figures 2A–C) with contrasting responses following RES compared to CON. Following RES, AP was significantly greater at 10 min compared to Rest, which was also greater than AER and CON (Figure 2A). Significant intervention-time interactions indicated greater AIx values following RES compared to AER and/or CON up to 40 min post-intervention (Figure 2B) with AIx75 values greater throughout the entire post-intervention period (Figure 2C). Additionally, AIx75 values following AER were greater than CON at 10 min post-intervention only. Following AER, AP, and AIx remained below Rest levels throughout the post-intervention with only the AIx value at 50 min reaching statistical significance (Figures 2A,B). In contrast, AIx 75 values for AER were elevated early post-intervention compared to Rest and declined to below Rest levels during the post-intervention period (Figure 2C). Following RES, AP, AIx, and AIx75 were significantly greater during early intervention with a gradual decline to near Rest levels for AP and AIx, but AIx75 values remained significantly elevated until 40 min post-intervention (Figure 2C). Following CON, values for AP, AIx, and AIx75 were lower throughout post-intervention compared to Rest (Figures 2A–C) with only AIx values reaching statistical significance (Figure 2B).

#### Cardiovascular responses

Significant intervention-time interactions confirmed greater HR values for RES compared to AER, which were greater than those for CON at most time points throughout the post-intervention period. Heart rates for RES and AER remained significantly greater than Rest values throughout the post-intervention (Table 2).

**Table 2:** Cardiovascular and reflection measures prior to and following aerobic (AER), resistance (RES), and no (CON) exercise interventions (*n* = 21).

		**Rest**	**10 min post**	**20 min post**	**30 min post**	**40 min post**	**50 min post**	**60 min post**	**Main effect of intervention**	**Main effect of time**
									***p* < 0.05**	***p* < 0.05**
**HR**	AER	56.3 ± 8.4	70.2 ± 8.3^*^^a^	65.9 ± 9.9^*^^a^	63.1 ± 8.6^*^^a^	63.3 ± 9.3^*^^a^	60.3 ± 7.4^*^	61.0 ± 7.4^*^^a^	RES>AER>CON	Rest < 10-60
(bpm)	RES	54.5 ± 5.9	89.6 ± 9.6^*^^†^^a^	83.8 ± 9.9^*^^†^^a^	78.2 ± 9.5^*^^†^^a^	75.0 ± 10.0^*^^†^^a^	71.0 ± 9.6^*^^†^^a^	68.4 ± 8.6^*^^†^^a^		10>30-60
	CON	54.6 ± 7.2	52.1 ± 6.2	53.0 ± 6.5	52.1 ± 6.2	52.3 ± 6.3	53.0 ± 7.5	51.9 ± 6.6		20>40-60
**bSBP**	AER	121.6 ± 11.2	123.6 ± 9.9	121.1 ± 10.8	120.2 ± 11.0	117.2 ± 10.8^a^	118.0 ± 8.8	118.5 ± 9.5		10>40
(mmHg)	RES	119.8 ± 13.2	125.1 ± 12.8^a^	122.1 ± 11.9	119.6 ± 10.6	119.4 ± 10.8	118.4 ± 9.2	120.0 ± 9.0		
	CON	122.0 ± 11.0	122.9 ± 9.8	124.4 ± 10.6	123.1 ± 8.6	123.2 ± 9.7	124.4 ± 11.3	124.8 ± 9.0		
**bDBP**	AER	69.3 ± 8.8	69.4 ± 8.4	68.0 ± 8.2	67.5 ± 8.6	66.3 ± 8.6^a^	66.6 ± 9.6^a^	68.8 ± 8.0	CON, AER>RES	Rest>30
(mmHg)	RES	67.5 ± 8.9	61.6 ± 9.3^*^^†^^a^	62.0 ± 7.4^*^^†^^a^	62.0 ± 7.8^*^^a^	61.8 ± 8.5^*^^a^	62.9 ± 8.0^*^^a^	64.4 ± 8.8^*^^a^		
	CON	67.7 ± 8.8	68.6 ± 8.8	68.8 ± 9.2	68.1 ± 10.5	70.1 ± 9.0	71.0 ± 9.8^a^	71.0 ± 9.5^a^		
**bMAP**	AER	86.8 ± 9.0	87.4 ± 8.1	85.8 ± 7.7	85.1 ± 8.1	83.3 ± 8.1	83.7 ± 8.3	85.3 ± 7.3	CON>RES	
(mmHg)	RES	84.9 ± 9.5	82.8 ± 8.9^**^†^**^	82.1 ± 7.0^*^	81.2 ± 6.9^*^	81.0 ± 7.5^*^	81.4 ± 7.2^*^	82.9 ± 7.7^*^		
	CON	85.8 ± 8.3	86.7 ± 7.7	87.3 ± 8.4	86.4 ± 8.7	87.8 ± 8.5	88.8 ± 9.3	88.9 ± 8.4		
**aSBP**	AER	108.5 ± 11.2	108.5 ± 9.4	106.8 ± 9.6	105.6 ± 9.6	103.2 ± 10.5	103.5 ± 8.9	104.9 ± 8.7		Rest>40
(mmHg)	RES	106.8 ± 12.3	110.7 ± 11.4	107.0 ± 9.9	104.9 ± 8.9	103.8 ± 9.3	103.0 ± 8.2	104.6 ± 8.5		10>30-60
	CON	108.8 ± 9.9	108.4 ± 8.6	109.3 ± 8.7	108.5 ± 8.4	109.0 ± 9.4	110.0 ± 11.0	109.7 ± 7.6		
**aDBP**	AER	70.6 ± 8.7	70.6 ± 8.2	69.7 ± 8.3	68.9 ± 8.4	67.4 ± 8.4	67.9 ± 9.4	70.1 ± 7.7	RES < AER, CON	
(mmHg)	RES	68.6 ± 8.9	63.7 ± 9.6	64.2 ± 7.1	64.6 ± 7.3	64.0 ± 8.1^*^	64.6 ± 8.1^*^	66.2 ± 8.4		
	CON	69.1 ± 8.5	69.7 ± 8.9	69.4 ± 9.5	70.7 ± 9.1	71.4 ± 8.9	72.1 ± 10.1	71.9 ± 8.9		
**aMAP**	AER	83.1 ± 10.7	86.0 ± 8.7	84.1 ± 8.4	82.9 ± 8.8	80.9 ± 8.9	81.0 ± 9.1	83.3 ± 8.6		10>30-50
(mmHg)	RES	82.0 ± 9.9	84.4 ± 10.3	81.8 ± 8.3	80.3 ± 8.3	79.5 ± 9.0	79.6 ± 8.4	81.1 ± 9.1		
	CON	83.0 ± 9.0	82.7 ± 8.4	83.1 ± 9.2	83.4 ± 8.6	84.2 ± 8.8	85.4 ± 9.8^a^	84.6 ± 8.7		
**Pf**	AER	26.4 ± 4.1	30.7 ± 4.7	29.0 ± 5.6	27.6 ± 4.5	28.0 ± 6.2	28.0 ± 6.2	25.9 ± 5.1	RES>AER, CON	10>Rest, 40, 60
(mmHg)	RES	26.6 ± 6.6	34.4 ± 8.6^a^	32.0 ± 7.2	30.1 ± 6.3	31.6 ± 6.0	30.5 ± 5.8	30.4 ± 6.1		
	CON	26.7 ± 5.6	26.9 ± 6.6	27.5 ± 5.8	26.6 ± 4.6	26.1 ± 4.1	26.7 ± 6.3	26.6 ± 4.2		
**Pb** (mmHg)	AER	16.0 ± 3.1	15.5 ± 3.2	15.0 ± 3.5	15.2 ± 2.4	14.3 ± 3.6	14.2 ± 3.0	14.2 ± 2.7	RES>AER, CON	10>40, 50, 60
	RES	16.0 ± 4.1	20.2 ± 4.5^*^^†^^a^	18.1 ± 3.6	16.8 ± 3.3	16.3 ± 3.4	16.0 ± 2.8	16.2 ± 2.8		
	CON	16.4 ± 3.9	15.3 ± 3.7	16.2 ± 3.8	15.7 ± 3.5	15.1 ± 3.0	15.0 ± 3.5	15.0 ± 2.6		
**RM**	AER	60.6 ± 12.6	50.6 ± 8.9	51.8 ± 10.3	55.3 ± 9.4	51.8 ± 12.3	51.5 ± 11.5	55.5 ± 11.0		Rest>40
(%)	RES	60.7 ± 15.4	60.0 ± 12.4	57.9 ± 13.6	56.9 ± 15.0	52.0 ± 10.2	52.7 ± 12.7	54.0 ± 9.4		
	CON	62.8 ± 14.8	57.7 ± 13.2	58.9 ± 10.9	59.2 ± 13.4	57.3 ± 11.8	57.2 ± 13.5	56.7 ± 11.6

*Data are expressed as mean ± SD; HR, heart rate; RES, resistance exercise; AER, aerobic exercise; CON, no exercise; bSBP, brachial systolic blood pressure; mmHg, millimeters of mercury; bDBP, brachial diastolic blood pressure; bMAP, brachial mean arterial pressure; bpm, beats per minute; aSBP, aortic systolic blood pressure; aDBP, aortic diastolic blood pressure; aMAP, aortic mean arterial pressure; Pf, forward wave; Pb, backward wave; RM, reflection magnitude*.

* *p < 0.05, significantly different to CON (intervention-by-time interaction)*.

† *p < 0.05, significantly different to AER (intervention-by-time interaction)*.

a *p < 0.05, significantly different to Rest within group*.

No significant intervention-time interaction or main effect of mode were identified for aortic systolic BP, but a main effect of time showed greater values at Rest and early post-intervention compared to late post-intervention (Table 2). For aortic diastolic BP, a significant intervention-time interaction demonstrated lower values following RES compared to CON during late post-intervention (Table 2). No significant intervention-time interaction effect was identified for aortic mean arterial pressure, but a main effect of time demonstrated significantly greater values at early (10 min) compared to late post-intervention (Table 2).

No significant intervention-time interaction or main effect of mode were observed for brachial systolic BP. However, a significant main effect of time identified values greater at 10 min compared to 40 min post-intervention (Table 2). Significant intervention-time interactions indicated lower brachial diastolic BP values following RES compared to CON throughout, and compared to AER in the first 20 min, post-intervention (Table 2). Values for brachial diastolic BP were significantly lower following RES compared to AER and CON and lower at 30 min compared to Rest (main effect). Significant intervention-time interactions identified lower brachial mean arterial pressure values for RES compared to CON at 20–60 min and AER at 10 min post-intervention (Table 2). Brachial mean arterial pressure values were significantly lower following RES compared to CON (main effect).

#### Reflection measures

A significant intervention-time interaction demonstrated greater Pb values following RES compared to AER and CON during early post-intervention (Table 2). Following RES, both Pf and Pb were significantly greater during early post-intervention compared to Rest with no significant changes following AER and CON (Table 2). No significant intervention or intervention-time effects were identified for reflection magnitude; however, a main effect of time identified greater values at Rest compared with those at 40 min post-intervention (Table 2).

## Discussion

To our knowledge, this was the first study to directly compare the influence of exercise mode (including a control group performing no exercise) on the time course of both arterial stiffness and pressure wave measures. A short bout of RES resulted in a significant increase in most parameters (AIx, AIx75, Pf, Pb) with AIx75 persistently elevated and greater compared to AER and CON throughout the 60-min, post-intervention period. These findings supported our hypothesis that RES would result in an acute increase in these measures. However, the hypothesized reduction in these measures with AER was not supported with non-significant changes in most measures (cf-PWV, Pf, Pb, and reflection magnitude) and a brief, significant increase in AIx75 early after the intervention. The current results highlighted the unique, acute changes in arterial stiffness and pressure wave measures following seated rest and exercise of different modes that exceeded their relevant technical error of measurement.

### Pulse wave velocity

Although not statistically significant (*p* < 0.1), our finding of elevated cf-PWV 10 min following RES was in line with earlier studies reporting increased cf-PWV following upper and whole body RES (Heffernan et al., 2007a; Fahs et al., 2009; Yoon et al., 2010) while others reported no effect following lower body RES (Heffernan et al., 2006, 2007b). Prior studies have suggested several processes modulating post-exercise cf-PWV with the exercise bout itself inducing significant changes in cardiovascular function that persist for some time after termination of exercise (Kingwell et al., 1997; Casey et al., 2008). As previously described (Pierce et al., 2018), several mechanisms have been implicated with the distinct BP adjustments during exercise, particularly during RES, likely playing a primary role for immediate changes in cf-PWV (e.g., 10 min post-exercise). However, this effect was not maintained throughout the post-exercise period with the absence of marked changes in both aortic and brachial systolic BP post-intervention likely linked to the minimal changes in post-exercise cf-PWV in the present study. It remains to be clarified if this transient increase in cf-PWV following RES simply reflects an acute response to exercise that subsides thereafter or if cumulative adaptations including pathological arterial wall modifications may result from repeat RES as chronic RES has resulted in increases (Okamoto et al., 2009), decreases (Au et al., 2017) and no change (Cortez-Cooper et al., 2008) in arterial stiffness

Our current results suggest minimal impact of acute AER on cf-PWV with prior studies reporting inconsistent results (Pierce et al., 2018). These prior contrasting findings were likely a result of different measurement techniques/systems (Rajzer et al., 2008), exercise protocols and/or participant cohorts (Zieman et al., 2005). Further, a recent review of short-term AER studies (Mutter et al., 2016) suggested that measurement of arterial stiffness and wave reflection responses immediately post-exercise (i.e., 0–5 min) commonly resulted in increased cf-PWV and/or AIx findings compared to later measurements (i.e., >5 min; Mutter et al., 2016). Further studies may elaborate upon the short-term alterations in these measures (<10 min) following AER and the potential mechanisms for such rapid adjustments that subside over time.

### Wave reflection measures (AIx, AIx75, Pf, Pb, reflection magnitude)

In the present study, whole-body RES resulted in significantly elevated and greater AIx, AIx75, Pf, and Pb compared to AER and CON, which continued throughout the post-intervention period for AIx75. In contrast, AER and CON resulted in similar AIx, Pf, Pb, and reflection magnitude with only a short-lived (10 min) significant increase in AIx75 following AER.

Few studies have previously investigated the effect of acute RES on wave reflection indices with contradictory results. In line with our findings, studies employing a protocol of either upper or whole-body RES reported increases in AIx and AIx75 (Fahs et al., 2009; Yoon et al., 2010), while studies employing a protocol of lower body RES reported decreases only (Rossow et al., 2012). The selective use of lower body muscle groups during AER in contrast to the inclusion of upper and lower body muscle groups during RES may have contributed to the distinct AIx/AIx75 responses with different exercise modes in our findings.

As previously described, wave reflection measures following exercise were moderated by multiple factors, including HR, the velocity (i.e., cf-PWV) and magnitude of the incident wave (i.e., Pf), left ventricular ejection duration, and arteriolar vasomotor tone (Kelly et al., 2001) from stimulation of the sympathetic nervous system (Okamoto et al., 2009). In the present study, both AIx and AIx75 were significantly elevated and greater following RES compared to AER and CON. With cf-PWV and Pf enhanced only briefly, this implicates arteriolar vasomotor tone as a major contributor to the enhanced wave reflection following RES. Greater perceptual and peripheral hemodynamic (i.e., RPE, HR, and BP) responses during RES compared to AER and CON in the present study supported the significant stimulation of the sympathetic nervous system, which continued throughout the post-intervention period. Peripheral vasoconstriction induced by the stimulation of the sympathetic nervous system with RES likely generated a significant increase in reflected wave intensity, as described previously (Kelly et al., 2001) with residual effects persisting throughout the post-intervention period. Although stimulation of the sympathetic nervous system presumably also occurred during AER, the vasoconstrictive effect may have been markedly reduced (Heffernan et al., 2007a) as distinct and exercise-mode dependent, shear stress patterns have been reported to affect vasoconstriction (Thijssen et al., 2017). Most studies to date, including the current study, have reported a reduction in AIx following AER (Kingwell et al., 1997; Heffernan et al., 2007a; Munir et al., 2008; Millen et al., 2016) with this result potentially linked to increased peripheral vasodilation rather than sympathetic nervous system-induced vasoconstriction. Vasodilation has been shown to occur in the presence of sympathetic activation with the endothelium-mediated vasodilator effect overriding the neural vasoconstrictor effect during AER (Piepoli et al., 1993). While a reduction in AIx may be considered beneficial for health, decreased AIx may simply reflect increases in HR (Wilkinson et al., 2000) rather than actual reductions in wave reflection. Therefore, correction for HR (i.e., assessing AIx75) may be crucial to examine exercise-induced changes in wave reflection that are masked by HR (e.g., Pf, Pb, reflection magnitude).

Like the wave reflection measures, reflection magnitude and its components have rarely been examined following exercise (Lefferts et al., 2014; Babcock et al., 2015; Millen et al., 2016). A decrease in post-exercise Pb, but not Pf, was reported immediately following 50 min of aerobic exercise at 60–75% of peak exercise (Millen et al., 2016) while an increase in Pf but not Pb was noted following upper body resistance exercise at 100% of 5-RM (Lefferts et al., 2014). In the current study, a greater post-exercise Pf and Pb were noted following RES only with Pb also greater compared to AER and CON. This result seems unrelated to post-exercise BP changes, as no significant and sustained increases in aortic or brachial BP were evident (Table 2). Differences in exercise modes and intensities, populations and timing of assessment make comparisons between the present and prior studies difficult, but the current results indicated that RES altered the magnitude of wave reflection, and that these were not associated with changes in central and peripheral BP, as suggested previously (Millen et al., 2016).

### Clinical implications

The relationship between exercise mode, arterial stiffness, and wave reflection measures is complex with the current results extending prior studies (Kingsley et al., 2016; Kobayashi et al., 2017) for a greater understanding of the physiological responses of acute exercise. A greater comprehension of these responses may assist in the selection of appropriate exercises for improved cardiovascular function, particularly for at-risk populations (e.g., hypertensive patients). The transient increase in wave reflection immediately following whole-body RES observed in this study may not be of significant concern in a young healthy population who exhibit normal resting levels as this acute response subsides within 1–72 h, as per current results. However, for at-risk populations in whom bouts of strenuous exercise are associated with acutely increased risk of cardiovascular events, the additional pressure imposed on the heart by augmented wave reflection resulting from whole-body RES may lead to an increased risk and/or incidence of a critical adverse event. Significant stimulation of the sympathetic-adrenergic system, post-exercise has been associated with an increased risk of myocardial arrhythmias (Del Rio et al., 2015), particularly in those susceptible to abnormal rhythms. Therefore, acute RES may amplify the risk for those with arrhythmia susceptibility or heightened sympathetic activity including chronic cardiovascular diseases (Parati and Esler, 2012). Medications, such as beta-blockers may provide some protection against this RES-induced risk (Hung et al., 2016) however, regular physical activity was reported to increase the sensitivity of beta-adrenergic receptors, thereby improving cardiovascular health (Santulli et al., 2013). Long-term adaptations within the beta-adrenergic system may be crucial to combat RES-induced changes in arterial function for high-risk populations that remains to be confirmed.

The exact timeline for the RES-induced elevated risk is not known as very few studies have examined wave reflection and/or arterial stiffness responses acutely following RES in clinical populations. However, long-term RES has been reported to increase resting cf-PWV by ~1.7 m·s^−1^ in hypertensive adults (Collier et al., 2008) and AIx by 9% in young women (Cortez-Cooper et al., 2005), changes likely associated with ~10–35% increased risk of a cardiovascular event and/or all-cause mortality (Vlachopoulos et al., 2010a,b). The elevated risk may be due to increased muscle sympathetic nerve activity associated with RES (Smith et al., 2015) with practitioners encouraged to consider carefully the use of RES for clinical populations. While the prescription of RES is actively encouraged for all populations due to its overall health benefits (Garber et al., 2011), practitioners may need to implement activities for the post-exercise period (e.g., cool-down) to minimize any negative changes in wave reflection and/or arterial stiffness. To our knowledge, the acute effects of combined AER and RES (i.e., concurrent training) on wave reflection and/or arterial stiffness measures have been rarely examined in clinical populations. Nevertheless, combined AER and RES chronic regimes have resulted in beneficial changes in resting wave reflection and/or arterial stiffness measures for older adults (Cook et al., 2006), women with metabolic syndrome (Eleuterio-Silva et al., 2013), obese men/women (Yang et al., 2011), patients with cardiovascular disease (Zhang et al., 2018) and hypertensive adults (Li et al., 2015; Jeon et al., 2018). The inclusion of AER following RES during an acute exercise session may counteract the potential negative effects of RES (Fahs et al., 2009; Sardeli et al., 2018; Tai et al., 2018) with acute AER reported to improve wave reflection and/or arterial stiffness measures in young (Munir et al., 2008; Lane et al., 2013; Milatz et al., 2015; Kobayashi et al., 2017) and older (Perissiou et al., 2018) healthy adults. Identifying both the acute and chronic responses of wave reflection and/or arterial stiffness in clinical populations could assist in establishing superior and safe exercise prescription for enhanced cardiovascular function and long-term health in these cohorts. Further, determination of the time kinetics of this post-exercise response, acutely and chronically with repeated exercise sessions, may clarify the potential benefits or susceptibility of arterial function to exercise, and the long-term link with health outcomes, morbidity and mortality.

### Limitations

For this preliminary study, exercise bouts were not matched for exercise intensity or volume but rather followed commonly prescribed bouts for cardiovascular health (Garber et al., 2011). The intention was to identify arterial stiffness and wave reflection responses to exercise prescribed for health maintenance and/or improvement. Future studies may compare different intensities and volumes within both exercise modes to explore the impact of these factors. Also, monitoring post-intervention arterial stiffness responses every 10-min for the first 60 min only may have resulted in a failure to identify subtle changes or those outside this initial period (e.g., 1–72 h). The current timeframe was selected and based on previous studies suggesting that cf-PWV returned to resting levels within 60 min post-intervention (Kingwell et al., 1997). Furthermore, all participants were recreationally active with these healthy responses potentially different to sedentary and/or clinical populations, which remains to be explored. Finally, the current study focused on a group of healthy, adult males which was powerful in terms of statistical analysis, compared to prior studies where sample sizes were <17 (Heffernan et al., 2007a; Kingsley et al., 2016; Kobayashi et al., 2017), but may somewhat limit the generalizability of our results to the entire general population. Studies have previously reported sex differences in both resting cf-PWV and cf-PWV following acute aerobic exercise (Doonan et al., 2013; Lane et al., 2013; Nieman et al., 2013; Baldo et al., 2018) with menstrual cycle phase potentially also affecting cf-PWV values (Madhura and Sandhya, 2014). Aiming to keep our participant cohort as homogenous as possible, the selection of a healthy, exclusively male cohort allowed the assessment of normal physiological responses to acute exercise in this specific population group without the influence of gender, substantial sex hormone variations and disease (Doonan et al., 2013). Our participants exhibited similar demographic characteristics to those in other studies (Yoon et al., 2010) for comparison while also representing an important cohort likely to experience future increased arterial stiffness and risk of cardiovascular disease (Gensini et al., 1996). The greater incidence of cardiovascular disease in men below 50 compared to women of similar age (Gensini et al., 1996) increases the relevance of our findings for those likely to suffer cardiovascular disease in the future. Prospective studies may extend our preliminary findings to females, who potentially exhibit differential arterial stiffness responses compared to males (Doonan et al., 2013) and those with clinical conditions (Yang et al., 2011; Eleuterio-Silva et al., 2013; Li et al., 2015; Jeon et al., 2018).

## Conclusions

The present study demonstrated that exercise mode influenced the time course of arterial stiffness and wave reflection responses to acute exercise in healthy adult males. Distinct hemodynamic and vasoconstriction responses seen with different exercise modes have been indicated as key modulators of cf-PWV and wave reflection. Indices of wave reflection should complement cf-PWV assessment to gain a more comprehensive picture of arterial function and left ventricular afterload. Inclusion of a control (no exercise) intervention and measures of technical error are highly recommended for future studies to ensure clear identification of significant changes in arterial stiffness, post-exercise.

## Author contributions

DP, KD, HR, and AL contributed to the design of this study, collection of data and data analysis. DP wrote the first draft of the manuscript and all authors contributed to interpretation of data, critical revision of the manuscript for important intellectual content and approved the final manuscript version.

### Conflict of interest statement

The authors declare that the research was conducted in the absence of any commercial or financial relationships that could be construed as a potential conflict of interest.
